# Preliminary Results on How Longer Facial Hair Lengths May Interfere With N95 Respirator Efficacy: A Brief Report

**DOI:** 10.1177/21650799241230039

**Published:** 2025-04-17

**Authors:** Vinicius da Eira Silva, Meagan Abele, Ian Bercovitz, Sherri Ferguson

**Affiliations:** 1 Department of Biomedical Physiology and Kinesiology, Simon Fraser University; 2 Environmental Medicine and Physiology Unit, Simon Fraser University; 3 Department of Statistics and Actuarial Science, Simon Fraser University

**Keywords:** beard, facial hair, N95, respirator fitting, seal check

## Abstract

**Background:**

The use of the N95 respirator outside work environments calls for a deeper understanding of the factors that interfere with its fitting, thus effectiveness. Here we determined how beard length influences N95 effectiveness. This research will improve guidance for individuals that use N95s in public spaces but cannot shave due to personal reasons.

**Methods:**

Bearded males (*N* = 28) participated in this study. Participants’ beard length was measured at the chin, mid jawline, and corner of the mouth, and a respirator fit tester was used to conduct a quantitative fit test. Participants then shaved and re-took the test. Fisher’s exact test was conducted to determine the association between bearded (BEA) and clean-shaven (CLE) conditions and test passing rate. A mixed effects model was conducted with participants as a random factor to determine the differences in fit factor (FF) scores between conditions. Finally, a regression analysis was completed to determine if there was a linear relationship between the FF response and beard length at the three locations.

**Findings:**

No statistically significant difference in passing rate (*p*-value = .79) and mean FF scores between BEA and CLE (*F*_1,54_ = 0.75, *p*-value = .39) was found. Although the regression analysis failed to detect a statistically significant relationship between the FF and beard length at the chin, mid jawline, and corner of the mouth (*p*-values = .07, .27, and .11, respectively), the results showed a decrease in FF scores when beard length increased.

**Conclusion/Application to Practice:**

Individuals who cannot shave completely should be encouraged to keep their beard as short as possible since beard length negatively impacts N95 effectiveness.

## Background

During the COVID-19 pandemic people started wearing masks in public places to protect themselves. The high protective factor of the N95 against the virus made it a popular option (Feng et al., 2020). In the workplace current standards and guidelines specify that N95 wearers must be clean-shaven during use to optimize fit, since facial hair may create a physical seal barrier between the N95 and the face (Occupational Safety and Health Administration [OSHA], 2006; Prince et al., 2021). The U.S. OSHA Respiratory Protection Standard (29 CFR 1910.134) requires employers to protect workers from respiratory hazards which includes providing respirators when needed and fit testing. Tight fitting respirators are not to be worn by employees who have facial hair or any conditions that interfere with face to facepiece seal or valve function (U.S. Department of Labor, Occupational Safety and Health Administration, 2006), and an alternate device or protection will be needed. However, regulating N95 use in public spaces presents unique challenges, as some individuals cannot shave due personal circumstances. To provide guidance for those individuals, it is important to better understand the impacts of beard length on N95 fitting, thus effectiveness. Yet, there are limited data on how different beard lengths may affect fit. The studies that investigated different beard lengths used visual standards to separate participants into categories instead of measuring the beard length (Sandaradura et al., 2020; Singh et al., 2020), investigated a narrow range of lengths (Prince et al., 2021), or used different individuals for the bearded and the controls, not accounting for differences in facial structures between groups (Fergin, 1984; Sandaradura et al., 2020; Skretvedt & Loschoavo, 1984). Considering these limitations in past research, this study aims to determine the effects of beard lengths on N95 fit. We hypothesized that increases in beard length would decrease N95 effectiveness.

## Methods

In this study the aim was to determine the impact of beard length on N95 effectiveness by using a quantitative fit test in repeated measures design to better control for facial structure differences between conditions. The quantitative Canadian Standards Association’s (CSA, 2002) fit test was performed on 28 adult male bearded participants (BEA) who later agreed to clean-shaving (CLE) and re-testing. Written informed consent was obtained from the participants for their anonymized information to be published in this article. All tests took place in a closed room with controlled temperature and humidity, 21.5°C to 25°C and 32% to 55%, respectively. A PortaCount 8026 Particle Generator (TSI, Inc., Shoreview, MN) was used to supplement the room with 0.05 μm diameter NaCl particles until it reached 9,000 particle counts/cm^3^. We waited 30 minutes after environmental conditions stabilized to bring the participants inside the room.

**Figure 1.**
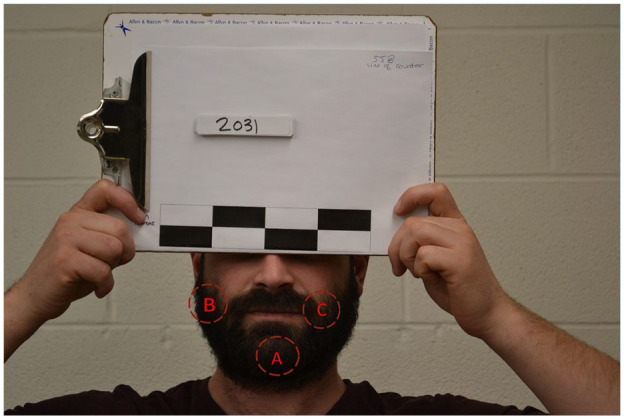
Beard measurement locations. (A) Chin (located at the mental protuberance of the mandible). (B) Mid jawline (located at the angle of the jaw). (C) Corner of the mouth (located at the labial commissure)

Before testing, participant’s beard length was measured in three places (Figure 1): chin (A- located at the mental protuberance of the mandible), mid jawline (B- located at the angle of the jaw), and corner of the mouth (C- located at the labial commissure). These areas were chosen as, despite the variation of beard styles, most involve hair growth in all the three locations.

For this study, participants chose between small or medium/large of N95 respirator sizes (Moldex 4200 N95p -Metric©, CA, USA) based on manufacture’s instructions. This model was selected because it is common among frontline workers and approved by the CSA and the National Institute for Occupational Safety and Health and it represents the sizes that fit most male faces (Zhuang et al., 2007). To ensure proper donning, participants performed a negative pressure seal check by cupping the N95 with both hands and sharply inhaling to create a noticeable negative pressure. If participants were unable to create negative pressure, they would redon their respirator or change the size until a proper seal was achieved. A steel sampling port was installed in each N95 using a TSI model 8025-N95 Fit Test Probe Kit, following manufacturer’s instructions regarding procedure and location, to allow sampling behind the mask. The participant’s N95 sampling port was then connected to a PortaCount Respirator Fit Tester 8038 (TSI^®^, MN, USA) using 10 feet of 0.25-inch conductive silicone tubing. An identical secondary tube was connected at the ambient sampling line. The system was set to continuously monitor ambient and behind mask particles in the size range 0.02 to 0.08 μm at a sampling rate of 1 per second. Participants then were cleared to start CSA’s fit test. The test consisted of seven tasks designed to simulate daily activities and encompassed a series of torso, head, and facial muscle movements (e.g., bending at the waist, reading aloud, turning head left and right, and up and down). Participants performed each task in order continuously for 1 minute before moving on to the next task. The fit factor (FF) was calculated as:

$$FF=\frac{Cb+Ca}{2Cr}$$

FF = fit factor; Cb = particle concentration in the ambient sample before the N95 sample; Ca = particle concentration in the ambient sample after the N95r sample; Cr = particle concentration in the N95’s sample.

Participants had to achieve a minimum FF score of 100 on each of the 7 tasks to pass the test (CSA, 2002). If the N95 received a failing grade on any task, participants had one more chance of re-size/re-fit the N95 and try again from the start. In case the N95 failed again, the test was ended and the latest FF score was computed as the result. After performing the bearded condition, participants would clean-shave and redo the exact same testing procedures a second time. Each participant’s results for both conditions were paired to determine the effects of beard length on the N95 fitting.

Fisher’s exact test of independence was calculated to determine if there was any association between passing rate and study arm (BEA vs. CLE). A mixed effects model was also calculated with participants as a random factor and study arm as a fixed effect factor to determine differences in FF scores between study arms, and a regression analysis completed to determine if there was a linear relationship between the FF response and beard length on the three measured locations. All procedures in this study were conducted in accordance with the principles embodied in the Declaration of Helsinki. The Office of Research Ethics of Simon Fraser University approved all the protocols (2019s0527).

## Results

Participants’ beard length varied between 1 and 70 mm at the chin (mean ± standard deviation [*SD*] = 17 ± 15 mm), 1 and 76 mm at the corner of the mouth (mean ± *SD* = 19 ± 17 mm), and 0 and 49 mm at jawline (Table 1, mean ± *SD* = 15 ± 13 mm). Fisher’s exact test of independence showed no significant association (*p*-value = .79) between groups and pass rate. The pass rates for the BEA and CLE were 47% (13 out of 28 analyzed) and 50% (14 out of 28 analyzed), respectively (Supplemental Table 1). The mixed effects model analysis failed to detect a statistically significant (*F*_1,54_ = 0.75, *p*-value = .39) difference in mean FF scores between study arms. The mean ± *SD*, FF scores for BEA and CLE were 89.9 ± 72.62 and 107.07 ± 75.03, respectively (Supplemental Table 2).

**Table 1.** Facial Anthropometric Measurements of Participants Mean, Median, Standard Deviation (STD), Minimum (Min), and Maximum (Max)

Variables (mm)	Participants (*N* = 28 males)				
	Mean	Median	STD	Min	Max
Beard length at chin	17	11	15	1	70
Beard length at the corner of mouth	19	12	17	1	76
Beard length at the jawline	15	12	13	0	49
Head circumference	581	580	17	540	610
Face length	121	121	8	102	136
Face width	140	138	4	132	151

**Figure 2.**
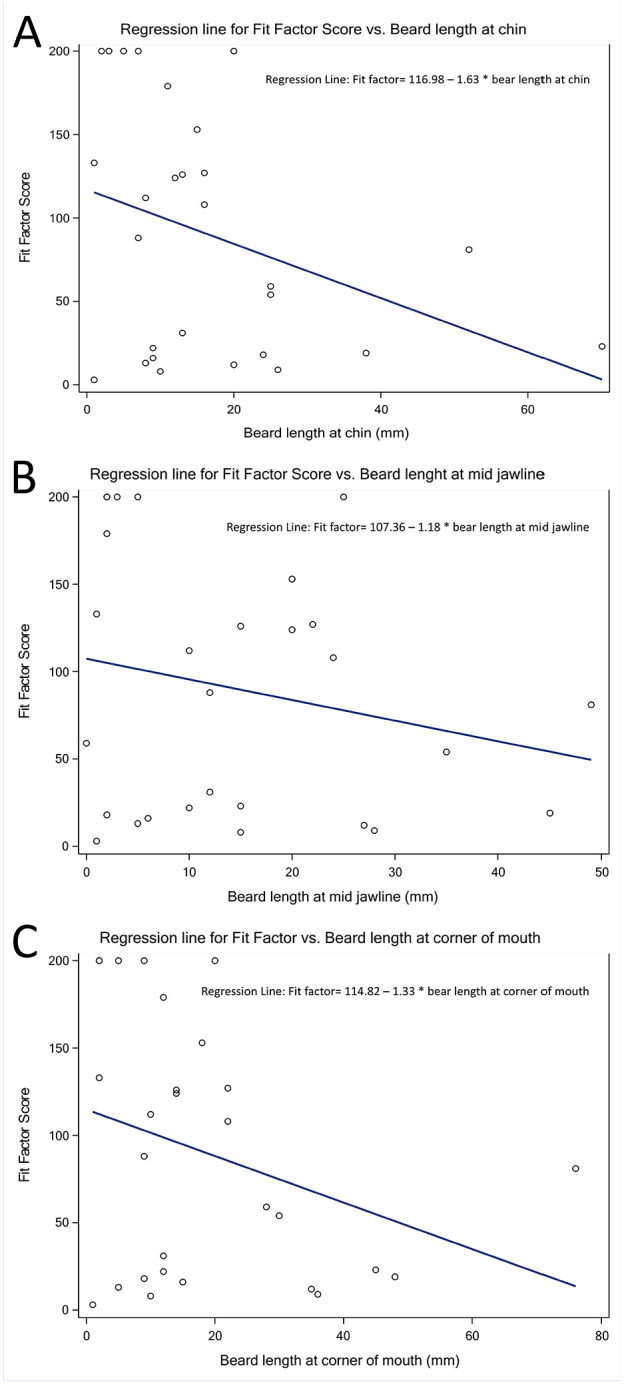
(A) Regression lines for Fit Factor (FF) versus Beard length at the chin. (B) Regression lines for Fit Factor (FF) versus Beard length at the mid jawline. (C) Regression lines for Fit Factor (FF) versus Beard length at the corner of the mouth

Lastly, the regression analysis failed to detect any statistically significant linear relationships between the fit factor response and length of hair at chin, jawline, and corner of mouth with *p*-values = .07, .27, and .11 respectively. The parameter estimates for slope were negative for all three independent variables, suggesting that FF scores decrease with increasing beard lengths. The slope estimates for length of hair at chin, jawline, and corner of mouth were found to be −1.63, −1.18, and −1.33 respectively (Figure 2, Panels A–C, respectively).

## Discussion

Previous studies that investigated the effects of beards on N95 performance reported inconsistent findings, with results ranging from severe (Skretvedt & Loschoavo, 1984) to moderate impairment (Prince et al., 2021). Comparing results between studies can be challenging as some studies did not measure participants’ beard length (Fergin, 1984; Sandaradura et al., 2020; Singh et al., 2020; Skretvedt & Loschoavo, 1984). Furthermore, some studies used different participants for the beard and control groups (Fergin, 1984; Sandaradura et al., 2020; Skretvedt & Loschoavo, 1984) which poses a severe limitation, as differences in facial shape between groups may play an important role in mask fit (Oestenstad & Bartolucci, 2010).

Contrary to a previous work by Sandaradura et al. (2020), the present study did not show significant differences in FF scores and passing rate between bearded and clean-shaven conditions. Although shaving increased the passing rate by one participant and the mean FF scores from 89.9 to 107.7 it was not enough to reach significance. The conflicting results between both studies might be caused by the difference in sample sizes. The sample size in this study was much smaller than the Sandaradura et al. (2020) study that used 105 participants. A larger sample size would be ideal in this study due to the large variability in our FF scores and dichotomic nature of the fit test (pass or fail). Future studies may want to replicate our design using a larger sample. Another possible explanation for the lack of significance in results was that only a single model of N95 was tested. Facial hair is only one of the facial attributes that can influence respirator fitting. Manufactures cannot account for every possible facial attribute when designing N95 models, meaning that some individuals will struggle more when trying to find a respirator that fits properly. Although all the N95s in this study passed the negative pressure test, a larger variety of N95 models and sizes would be ideal to help participants to find an optimal fit. In U.S. workplaces, employers must adhere to the OSHA Respiratory Protection Standard regarding respirator selection and usage and fit testing (U.S. Department of Labor, Occupational Safety and Health Administration, 2006).

The regression analysis showed a decrease in FF scores with longer beard lengths. Previous studies also demonstrated similar trends (Prince et al., 2021; Sandaradura et al., 2020). The strongest negative linear relationship (slope = −1.63) was found between FF scores and the beard in the chin area. N95 wearers in public spaces should be aware of the detrimental effects on fit with longer beards and, if not able to fully shave, minimize beard length specially around the chin area.

This study had three main differences when compared to previous studies: The first was the use of repeated measures design to investigate the effects of beard length on fit factor scores. That allowed for better control for facial differences between groups. Second, beard length was measured in three different locations, which allowed us to study the effects of facial hair location on FF scores. Finally, a larger range of beard lengths was investigated in this study (0–76 mm), when compared to a similarly designed study (0–10 mm; Prince et al., 2021).

### Limitations

The present study also contains limitations. First, the model of N95 used in this experiment only had two sizes available (small and medium/large). Although all participants achieved proper seal with the N95 model, using a greater variety of models and sizes might have increased the passing rates. Future studies may also try having a sub-sample of the participants to do the CLE condition first, with participants returning later for the BEA condition, to investigate the effects of condition order on passing rates. Another limitation was sample size, and due to the nature of measures used, the sample size might have been insufficient to reach significance. Future studies should try to replicate this study with a larger sample.

## Conclusion

This preliminary study shows a tendency of decrease in N95 fit with increase in beard length. This effect seems more pronounced when the beard is in the chin area. Additionally, achieving good fit with an N95 is challenging with or without facial hair, and fit testing for the general public should be performed whenever possible. In the U.S. workplace, respirator usage, and fit testing is governed by OSHA (29 CFR 1910.134).

**Applications to Professional Practice**

The COVID-19 pandemic changed our understanding of how vulnerable we are to viral transmission, and thus how we behave within public spaces to mitigate our newfound sense of risk. With the disease still not fully controlled and the promise of new variants and new pandemic waves in the future, the use of masks to protect against transmission will continue to be commonplace. Understanding how the length of beards—not just their presence—can affect the fit of N95s may help to develop new public policies and guidelines to better protect individuals in public settings during high transmission periods. Preliminary study data points out that longer beards, especially in the chin area, may interfere with N95 protection, leaving individuals more exposed to airborne viruses. Data from this study also demonstrated that achieving an acceptable mask fit and fit testing is indeed complex particularly for the public at large. Occupational health professionals can provide information to workers about the publics’ use of N95s and the importance of fit testing to achieve a proper face seal. Workers who do not require respiratory protection in the workplace can use this information for self-protection and also share the information with family and others. Lastly, the authors would like to acknowledge that this paper does not provide evidence to justify the presence of beards in work environments where there are regulations in place for respiratory protection provided by OSHA, such as healthcare.

## Supplemental Material

sj-docx-1-whs-10.1177_21650799241230039 – Supplemental material for Preliminary Results on How Longer Facial Hair Lengths May Interfere With N95 Respirator Efficacy: A Brief Report Supplemental material, sj-docx-1-whs-10.1177_21650799241230039 for Preliminary Results on How Longer Facial Hair Lengths May Interfere With N95 Respirator Efficacy: A Brief Report by Vinicius da Eira Silva, Meagan Abele, Ian Bercovitz and Sherri Ferguson in Workplace Health & Safety
