# Supplementary material for: Preliminary Results on How Longer Facial Hair Lengths May Interfere With N95 Respirator Efficacy: A Brief Report
Source: Workplace Health Saf. 2025 Apr 17;73(7):358–62. doi: 10.1177/21650799241230039 (PMC12099016; doi:10.1177/21650799241230039)
Supplement: sj-docx-1-whs-10.1177_21650799241230039 – Supplemental material for Preliminary Results on How Longer Facial Hair Lengths May Interfere With N95 Respirator Efficacy: A Brief Report [file sj-docx-1-whs-10.1177_21650799241230039.docx]

Supplementary material - da Eira Silva et al., Preliminary results on how longer facial hair lengths may interfere with N95 respirator efficacy: A brief report.

| **Pass rate for BEA and CLE study arms** | | | |
| --- | --- | --- | --- |
| **Study arm** | **Pass rate** | | |
| **Frequency (n) Row percentage (%)** | **Fail** | **Pass** | **Total** |
| **Beard (BEA)** | 15 53.57 | 13 46.43 | 28 |
| **Clean shaven (CLE)** | 14 50.00 | 14 50.00 | 28 |
| **Total** | 29 | 27 | 58 |

Online supplementary material table S1. Pass rate frequency and row percentage for beard (BEA) and clean shaven (CLE) study arms.

| **Study arm** | **Overall Fit Factor (FF)** | | | | | |
| --- | --- | --- | --- | --- | --- | --- |
|  | **N** | **Mean** | **Median** | **Std** | **Min** | **Max** |
| **Beard (BEA)** | 28 | 89.93 | 84.50 | 72.62 | 3.00 | 200.00 |
| **Clean shaven (CLE)** | 28 | 107.07 | 99.50 | 75.03 | 0.00 | 200.00 |

Online supplementary material table S2. Overall fit factor (FF) scores analyzed sample sizes, means, medians, standard deviation (Std), Minimum (Min) and Maximum (Max) for bearded (BEA) and clean shave (CLE) study arms.
